# Longitudinal Structural and Microvascular Observation in RCS Rat Eyes Using Optical Coherence Tomography Angiography

**DOI:** 10.1167/iovs.61.6.54

**Published:** 2020-06-24

**Authors:** Bingyao Tan, Veluchamy A. Barathi, Emily Lin, Candice Ho, Alfred Gan, Xinwen Yao, Anita Chan, Damon W.K. Wong, Jacqueline Chua, Gavin S. Tan, Leopold Schmetterer

**Affiliations:** 1 Singapore Eye Research Institute, Singapore National Eye Centre, Singapore; 2 SERI-NTU Advanced Ocular Engineering (STANCE) Program, Nanyang Technological University, Singapore; 3 Academic Clinical Program, Duke-NUS Medical School, Singapore; 4 Department of Ophthalmology, Yong Loo Lin School of Medicine, National University of Singapore and National University Health System, Singapore; 5 Department of Ophthalmology, Lee Kong Chian School of Medicine, Nanyang Technological University, Singapore; 6 Department of Clinical Pharmacology, Medical University of Vienna, Austria; 7 Center for Medical Physics and Biomedical Engineering, Medical University of Vienna, Austria

**Keywords:** optical coherence tomography angiography, vascular imaging, retinal imaging, choroidal imaging, retinal degeneration

## Abstract

**Purpose:**

To evaluate the change of retinal thickness and ocular microvasculature in a rat model of retinitis pigmentosa using swept source optical coherence tomography angiography (SS-OCTA)

**Methods:**

Three-weeks-old Royal College of Surgeons (RCS) rats (*n* = 8) and age-matched control rats (*n* = 14) were imaged by a prototype SS-OCTA system. Follow-up measurements occurred every three weeks on six RCS rats until week 18, and cross-sectional measurements were conducted on control rats. Thicknesses of different retinal layers and the total retina were measured. The enface angiograms from superficial vascular plexiform (SVP) and deep capillary plexiform (DCP) were analyzed, and the image sharpness was also extracted from the choroidal angiograms. Immunohistochemical analysis was done in the RCS rats after week 18, as well as in three-week-old RCS rats and age-matched controls.

**Results:**

In RCS rats, the thicknesses of the ganglion cell complex, the nuclear layer, the debris/photoreceptor layer and the total retina decreased over the weeks (*P* < 0.001). The SVP metrics remained unchanged whereas the DCP metrics decreased significantly over the weeks (*P* < 0.001). The immunohistochemical analysis confirmed our OCTA findings of capillary dropout in the DCP. The choroidal plexus appeared indistinct initially due to scattering of light at the intact retinal pigment epithelium (RPE) and became more visible after week nine probably due to RPE degeneration. Loss of choriocapillaris was visualized at week 18. In control rats, no vascular change was detected, but nuclear layers, photoreceptor layers and total retina showed slight thinning with age (*P* < 0.001).

**Conclusions:**

Photoreceptor degeneration in RCS rats was associated with the loss of capillaries in DCP, but not in SVP. The OCTA imaging allows for the characterization of structural and angiographic changes in rodent models.

Retinitis pigmentosa (RP) is a vision threatening disease that affects 1/3500 to 1/4000 people in the United States and Europe.^1^^,^^2^ It is characterized by the defective mutation of more than 50 genes affecting retinal pigment epithelium (RPE) and photoreceptors.^3^^,^^4^ Resultant symptoms typically appear in childhood, including night vision difficulties and loss of peripheral vision. The clinical assessment of RP focuses on detecting the function of photoreceptors by electroretinograms^5^^–^^8^ and the structure of the retina by optical coherence tomography (OCT).^9^^–^^14^ Recent studies also indicated insufficient metabolic and oxygen supply to the photoreceptors that could trigger secondary sensory degeneration.^15^^–^^18^ Furthermore, retinal and choroidal vessel attenuation was observed in patients with RP using fluorescein angiography (FA),^19^ and deep capillary plexiform (DCP) vasculature was reported to be more affected than superficial vascular plexiform (SVP) vasculature in RP patients using depth-resolved OCTA.^20^ So far these studies only involved cross-sectional RP participants and little is known about the relationship between the progressive dystrophy of the photoreceptors and their nourishing microvasculature environment.

The Royal College of Surgeons (RCS) rat is the first inherited dystrophic model of retinal degeneration characterized by progressive photoreceptor degeneration,^21^ and it has been extensively used as a model to study RP.^22^^–^^30^ A mutation of *mertk* gene leads to rod cell loss by preventing the RPE from phagocytizing and disc shedding, and causes an accumulated debris layer in the photoreceptor layers. Since its development in 1962 by Sidman,^21^ numerous studies have been conducted to study the progression of functional and structural change of the retina using ERG ^24–26,31^and OCT.^22^^,^^24^^,^^31^^,^^32^ However, previous microvascular studies were cross-sectional because of a lack of noninvasive imaging tools. Recently, fundus FA was used by Shen and colleagues^33^ to monitor the retinal vasculature over time and progressive vascular leakage was reported, but this approach had no depth resolution for differentiating vascular plexuses. Moreover, it provided no insight into choroidal vessels as they were partially blocked by RPE and appeared as a diffuse bright background.

OCT Angiography (OCTA) is an extension of OCT that uses high-speed image acquisition to conduct repeated scans at the same position, and a decorrelation calculation algorithm extracts the motion contrast from the cross section, namely the blood vessels.^34^^–^^36^ OCTA has been used as a powerful tool to study rat models of microvascular ocular diseases, such as diabetic retinopathy,^37^ ocular hypertension,^38^^,^^39^ and choroidal neovascularization.^40^^,^^41^ It is a noninvasive imaging modality that offers high-resolution three-dimensional information of the vasculature without the need for exogenous dye injection and is less time-consuming than FA. Swept source OCTA uses a spectrum-scanning laser as the light source and the spectral fringes are detected by a single photodetector. Compared to the spectral domain OCT, it has better signal roll-off and is usually associated with higher tissue penetration by using longer wavelength lasers. SS-OCTA showed advances over SD-OCTA in imaging deep tissues, such as laminar cribrosa,^42^^,^^43^ choroid^44^ and even sclera.^45^^,^^46^

Based on the considerations above, we performed a longitudinal study in the RCS rat model using SS-OCTA. Structural and angiographic information were extracted from the retina and the choroid. Quantitative analysis was done to investigate the retinal thickness and microvascular change over the weeks, as compared to age-matched controls. Whole-mount immunohistochemistry was also conducted to verify vascular changes.

## Methods

All procedures in this study were conducted in accordance to the ARVO Statement for the Use of Animals in Ophthalmic and Vision Research. The study protocol was approved by the Institutional Animal Care and Use Committee of SingHealth (Singapore). Animals were kept in a 12/12 hours light/dark illumination-controlled room, and water and food were fed ad libitum. They were separated into three groups (Table): Group I (Albino RCS was purchased from Kyoto, Japan, lab bred, *n* = 2) was sacrificed at week 3 and served as baseline immunohistochemistry group. Group II (Albino RCS, lab bred, *n* = 6) was imaged by OCTA every three weeks from three-week postnatal, followed by immunohistochemistry at week 18. Group III (Albino Wistar Hannover GALAS was purchased from In-Vivos, Singapore, lab bred, *n* = 14) was a cross-sectional sample of rats with a variety of ages (3–18 weeks) serving as a control group. Animals from group III were imaged once using OCTA, and five of them were killed for immunohistochemistry.

**Table.** Details of Experimental Groups

Group	Animal Type	Sample Size	Age (Weeks)	Procedure
I	RCS	2	3	Baseline Immunohistochemistry
II	RCS	6	3–18 (Follow-up)	OCTA/Immunohistochemistry
III	Wistar	14	3–18 (Cross-sectional)	OCTA/Immunohistochemistry

### Optical Coherence Tomography Angiography

Before the experiment, ketamine/xylazine (ketamine: 0.08 mL/100 g; xylazine: 0.05 mL/100 g) was delivered intraperitoneally for anesthesia, and rats’ breathing was monitored closely until the rate stabilized. One drop of tropicamide (1.0%; Alcon, Singapore) was administrated on each eye for pupil dilation, and a zero-diopter contact lens (Cantor & Nissel, Brackley, United Kingdom) was placed on the imaged eye. The whiskers were cut off to ensure a clear window of imaging. During the experiment, the breathing rate was monitored frequently, and artificial tears were applied every five minutes.

A prototype SS-OCTA (PlexElite 9000; Zeiss Meditec, Dublin, CA, USA) was used to image the rat retinas. Briefly, it uses a wavelength scanning laser (central wavelength, λ_c_ = 1050 nm; bandwidth, ∆λ = 100 nm) as the light source, and the spectral information is acquired by a photodetector. The system operation speed is 100,000 Ascan/second, and the axial and lateral resolution in tissues are 6.3 µm and 5 µm when used in the rat eye, respectively. A built-in wide-field 15 × 9 mm^2^ scanning protocol was applied, but notably the scanning area was with reference to the size of the human eye (axial length: ∼24 mm), and the actual scanning area in the rat eye was about 3.9 × 2.4 mm^2^, assuming an axial length of 6.3 mm.^47^ Each volume was sampled by 800 B-scans where each B-scan consisted of 500 A-scans, and B-scans were repeated two times. An optical microangiography protocol was used to calculate the depth-resolved angiographic information.^48^ A customized animal holder with five degrees of freedom (3 translational + 2 rotational) was used for eye alignment.

### Post Processing and Image Analysis

The retinal layer segmentation was done automatically by a review software (PlexElite Review Software) to separate the superficial vascular plexus (inner limited membrane–ganglion cell layer), the deep vascular plexus (inner plexiform layer–outer plexiform layer), and choroidal plexus (retinal pigmented epithelium [RPE]–100 µm below RPE). Manual correction was applied wherever necessary to ensure appropriate layer segmentation. Several enface angiograms were generated by taking the vertical maximum projection in each slab, and the projection artifact from the superficial vessels was removed automatically. These angiograms were exported to MATLAB (MathWorks Inc, Natick, MA, USA) for further analysis.

**Figure 1.**
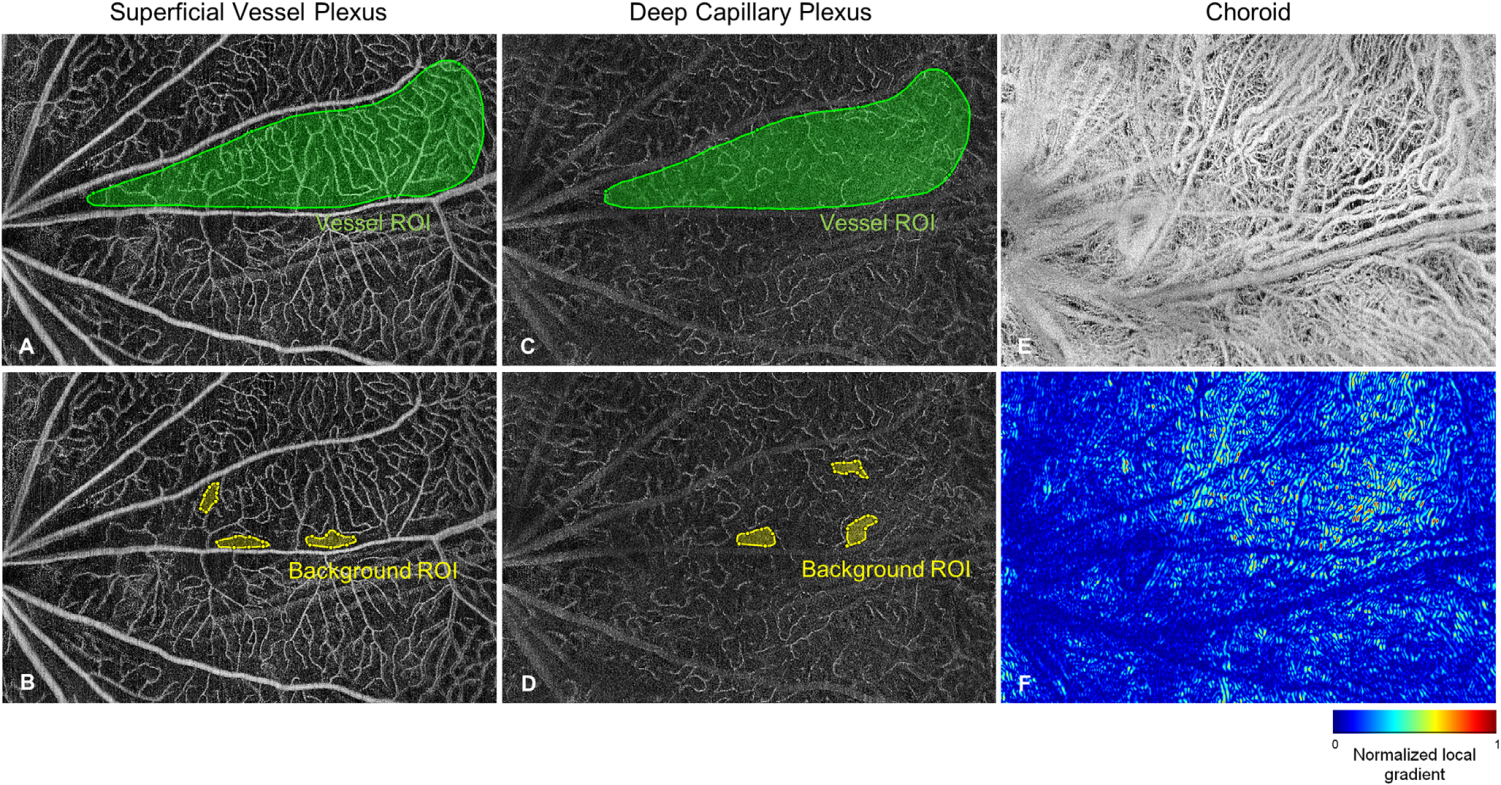
OCTA quantification on the retinal plexuses and the choroidal plexus.

To quantify the retinal vessels, the OCTA images were first smoothed by a 3- × 3-pixel median filter. The image quality over the entire field was not homogenous probably due to optical vignetting or motion artifacts. Therefore only the regions of interest (ROIs) between the major vessels that showed good contrast and sharpness were selected for vessel analysis. For the inner retinal vasculature, vessel index was calculated as the intensity difference between the vessels and the background. Specially, a manual region selection function (*drawfreehand*, MATLAB) was used to select a vessel ROI (green regions in Figs. 1A, 1C) and multiple intercapillary background ROI (yellow regions in Figs. 1B, 1D). Then the vessel index was calculated as:

$$\mathrm{Vessel}\;\mathrm{Index}\left(\mathrm{VI}\right)=\frac{\sum_{\mathrm{ROI}}I_{i,j}}{A_{\mathrm{ROI}}}-n_{\mathrm{bkgROI}},\;\left(i,j\right)\in \mathrm{ROI}$$

Here, *I*_*i*,*j*_ denotes the intensity of a pixel at (i,j) location, A_ROI_ denotes the area of selected vessel ROI, and *n*_bkgROI_ denotes the mean intensity of the background ROI.

For the choroidal plexus, the quantification of the choroidal vessel alteration is challenging due to the dystrophy of the RPE layer, which progresses over time. The RPE, however, is a highly scattering structure and the choroid is not clearly visible in healthy animals. With the RPE getting destroyed over time, the visibility of the choroid gets better, a phenomenon well known from imaging patients with geographic atrophy.^49^ Therefore we investigated the sharpness of the choroidal plexus as an indicator of scattering potential of the RPE, and the sharpness of choroidal plexus was calculated as the sum of the local gradient values over the entire gradient image:

$$\alpha =\sum_{i=1}^{N_{x}}\sum_{j=1}^{N_{y}}\left|\nabla \left(G_{3}*I_{i,j}\right)\right|$$

where G_3_ is the Gaussian convolution with a kernel size of 3 × 3 pixels, ∇ denotes the partially derivative and N_x_, N_y_ denote the numbers of pixels in vertical and horizontal directions. Examples of choroidal plexus and their corresponding gradient image were shown in Figs. 1E-F.

The retinal layer thickness was measured manually from two representative B-scans approximately 1 mm and 2 mm away from the ONH, respectively, where the ONH location was determined from the enface view of the entire retina. On each B-scan, two locations were selected for thickness measurement, and the thickness values from two B-scans were averaged. The manual retinal segmentation was based on clear boundaries with large gradient change in the vertical direction.^50^

### Immunohistochemistry

After scarification of the rats, the whole rat eye was enucleated and fixed in 4% paraformaldehyde (PFA) (Sigma-Aldrich, St. Louis, MO, USA) for 2 hours. The eye was then dissected into anterior and posterior segment. Anterior segment of the eye was discarded leaving only the posterior eye cup. The retina was then carefully dissected from the sclera and the choroid, and care was taken to prevent any incision while being submerged in cold 70% ethanol on a petri dish. The retinas were then transferred to a 24-well plate for subsequent enzymatic and antibodies treatment. Initially they were enzymatically treated with collagenase/dispase for one hour (2 mg/mL in phosphate-buffered saline solution [PBS]; Roche 269 638). Then they were washed three times for five minutes each with PBS and nonspecific sites were blocked with blocking solution of 10% goat serum and 1% Triton X-100 in PBS for four hours at room temperature. After three days of incubation, corresponding secondary antibodies - Alexa Fluro 488/594 – conjugated fluorescein-labeled secondary antibody (1: 500; Invitrogen Molecular Probes, Eugene, OR, USA) and conjugated blood vessel marker Isolectin GS-IB4-647 (1:75; Invitrogen) were applied and incubated for a further 2 days at 4°C shaking on a low-speed orbital shaker. The slides were then washed twice with PBS and once with PBS with 0.1% tween for 10 minutes each and mounted on Polysine microscope glass slides (Gerhard Menzel; Thermo Fisher Scientific, Newington, CT, USA). A confocal microscope system (FV3000, Olympus, Tokyo, Japan) was used to examine the slides and high-resolution images (×20) were captured.

### Statistics

Two-way repeated measures analysis of variance (ANOVA) was used to analyze within-animal changes in retinal layer thickness, vessel index, and choroidal vessel sharpness index over time, whereby week and eye were included as repeated variables in the model. The Greenhouse-Geisser method was used to adjust the *F*-test of the within-animal effect of week for nonsphericity. Post-hoc pairwise contrasts between consecutive weekly measurements were estimated and *P* values were corrected for multiple testing using Bonferroni's procedure. To assess whether there was a monotonically increasing or decreasing trend with time, we also conducted a post-hoc test of the linear orthogonal contrast following ANOVA, the result of which is referred to as *P* trend in the results. One-way ANOVA was used to compare measurements between rats of different age groups in the control group, with post-hoc pairwise comparisons likewise corrected for multiple testing. A *P* value < 0.05 was regarded as statistically significant.

## Results

**Figure 2.**
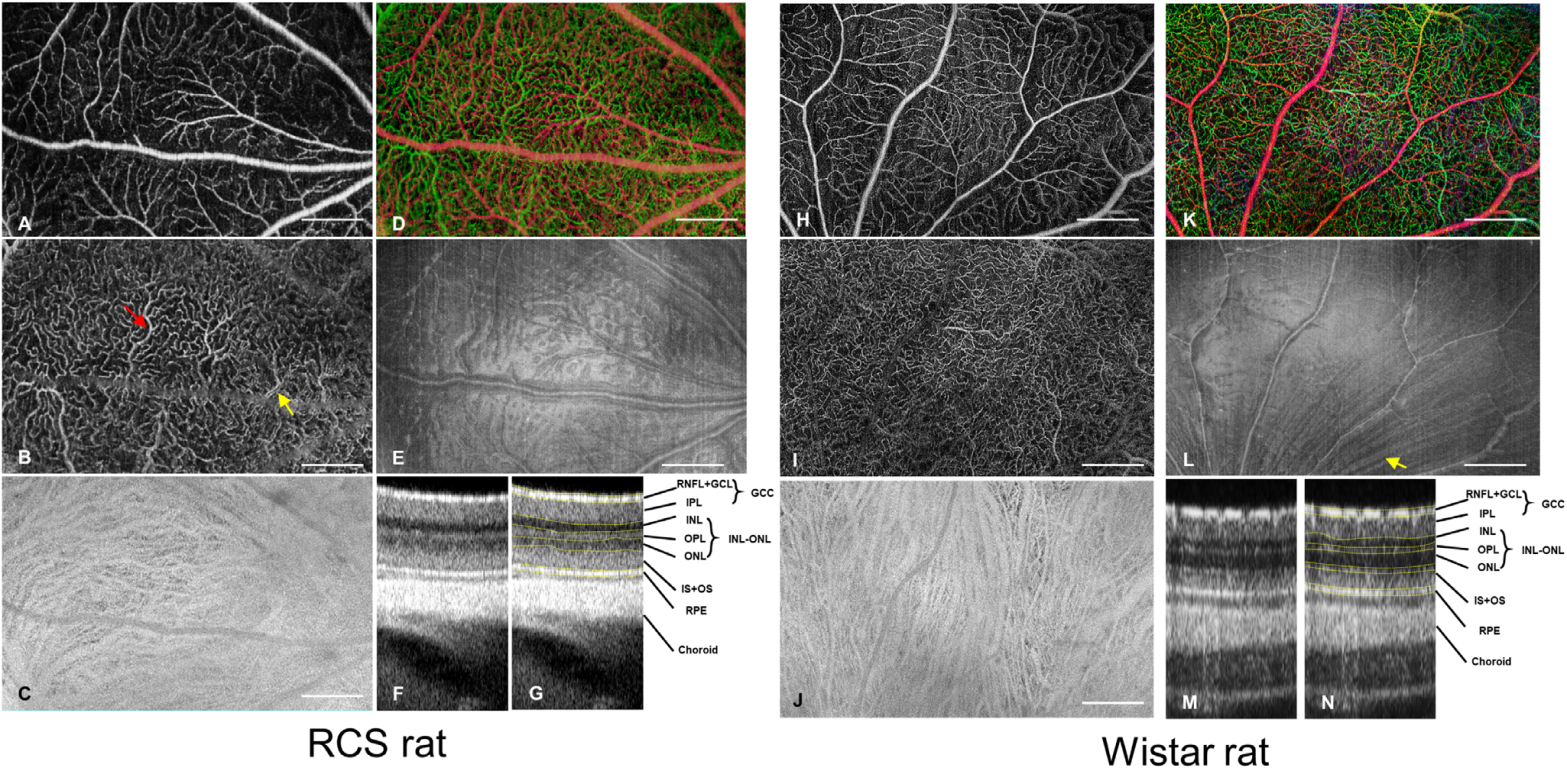
Representative structural and angiographic images from one RCS rat (age three weeks) and one Wistar rat (age six weeks). (**A,H**) Superficial vascular plexus. (**B,I**) Deep vascular plexus. (**C,J**) Choroidal plexus. (**D,K**) Depth coded retinal vasculature. *Red*: Superficial vascular plexus, *Green*: Deep vascular plexus. (**E,L**) structural image from retinal nerve fiber layer (RNFL). The hyperreflection is a consequence from surface reflection of inner limited membrane and vessel wall of major blood vessels. (**F,G,M,N**) Cross-sectional OCT scans showed distinguishable retinal layers, with and without manual segmentation. External limited membrane was not visible from RCS rat retina. IPL, inner plexiform layer; IS, inner segment; OS, outer segment. *Red arrow*: capillary that was on focus and appeared sharp. *Yellow arrow*: double-vessel appearance probably due to optical vignetting or motion. *White arrow*: nerve fiber bundles. Scale bar: 500 µm.

Wide-field images from one RCS rat eye at week 3 and one control rat eye at week 6 are demonstrated in Figure 2. The enface angiograms from the superficial (A,H), deep (B,I) and choroidal plexuses (C,J) clearly showed the structure of the microvasculature. The red arrow labeled a capillary that shows high contrast, whereas the yellow arrow labeled a capillary showing dual lines and low contrast probably due to optical vignetting or motion. Figures 1D and 1K show the depth encoded OCTA images, where the red color represents the SVP and the green color represents the DCP. *The enface* morphologic images from the superficial layer (Figs. 1E, 1L) showed surface reflection from the inner limited membrane (ILM) and the embedded large vessels. Cross-sectional images of the retina with distinguishable individual retinal layers are shown in Figures 1F, 1G and Figures 1L–1N. Uncompensated dispersion was noticed on these cross-sectional scans because we did not have access to the spectral data, which slightly blurred the image in the vertical direction. External limited membrane (ELM) was distinguishable in the control rat retina but not in the RCS rat retina, probably due to the photoreceptor change before week 3.

**Figure 3.**
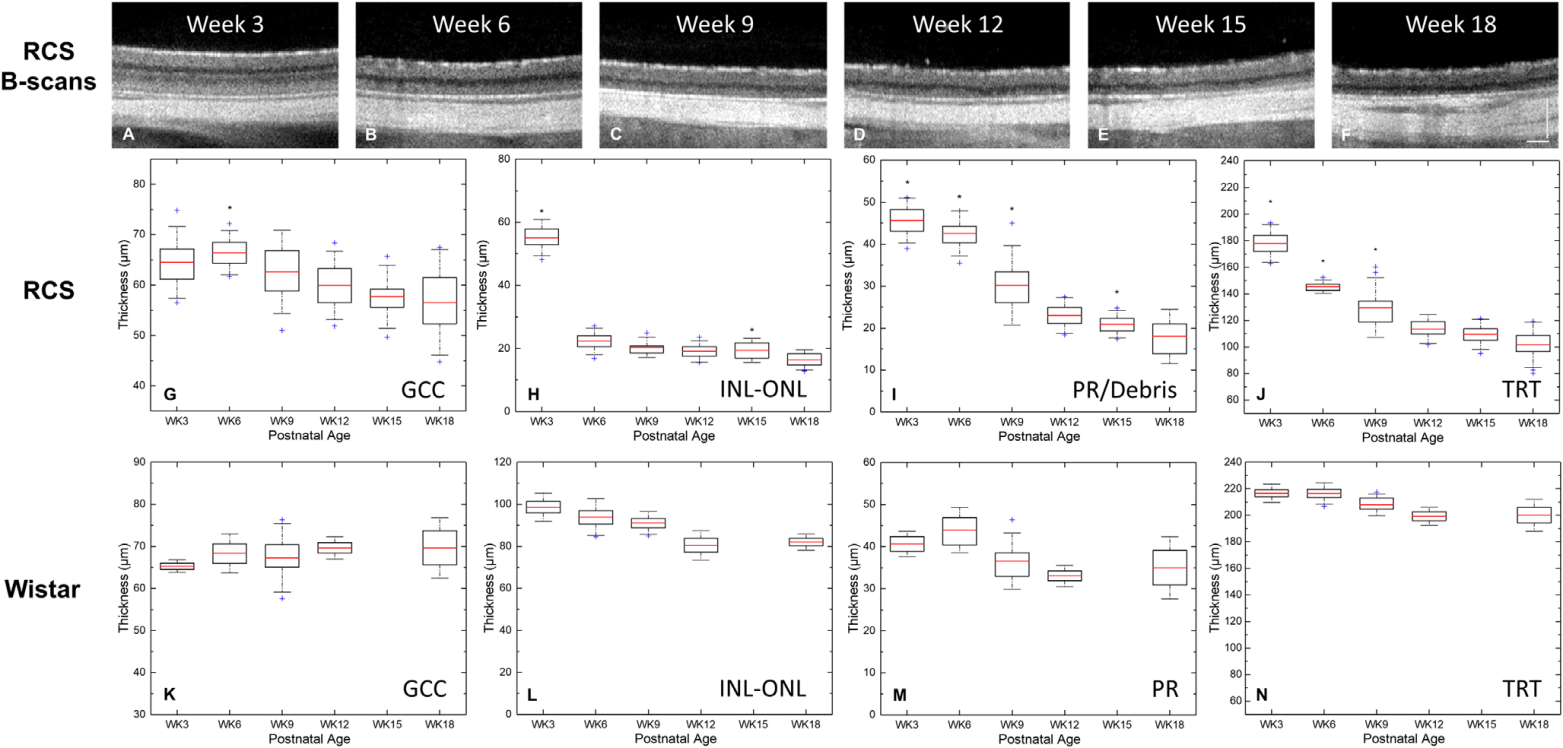
Summary of retinal thickness measurement. (**A–F**) Representative cross-sectional OCT images from ∼1 mm from the optic nerve head. OPL was still distinguishable at week 3, but visibility was lost from week 6 on. Boxplot of the retinal thickness from RCS rats and the age-matched Wistar rats are shown in **G–J** and **K–N**, respectively. *Red bar*: mean value. Box edge: 25 and 75 percentiles. Whisker: 1.5 × standard deviation (SD). TRT, total retinal thickness. *Significant thickness difference from the proceeding measurement. Significant difference: *P* < 0.05. Scale bar = 500 µm.

The progressive thinning of the retina was observed from cross-sectional scans (Figs. 3A–3F). Visually, the outer plexiform layer (OPL) and outer nuclear layer (ONL) were still separable at week 3 but could not be distinguished from week 6 onward. The intensity from the choroid layer gradually became brighter from week 3 to 9, probably due to dystrophy of the RPE layer. Hence, fewer photons were scattered from this dense optical diffuser. The quantitative analysis is summarized in Figures 2G–2N. Retinal nerve fiber layer (RNFL), ganglion cell layer (GCL) and inner plexiform layer were combined as ganglion cell complex (GCC) because RNFL+GCL are thin and difficult to measure separately, and inner nuclear layer (INL), OPL and ONL were combined as one nuclear layer (INL-ONL) as they were inseparable in RCS rats after week 6. In RCS rats, progressive thinning of GCC, INL-ONL and photoreceptor (PR)/debris layers were detected over time (all *P* < 0.05, *P* trend). Specifically, GCC underwent a total thinning of 12.1% ± 10.8% from week 3 to 18. For INL-ONL layer, there was a rapid thinning of 69.3% ± 6.4% from week 3 to week 6 (*P* < 0.001), followed by a relatively slow decrease until week 18, which was, however, not significant (all *P* > 0.123), expect between week 15 and 18 (*P* = 0.007). The PR/debris layer showed a slow thickness reduction of 6.4% ± 10.7% from weeks 3 to 6 (*P* = 0.024), followed by a rapid thinning of 45.2% ± 11.6% till week 12 (both P < 0.001). It was then stabilized till week 18. The total retinal thickness measured from ILM to RPE showed a continuous decrease over the weeks (*P* < 0.001). Similar to the nuclear layer, the thinning was sharp and significant between measurements till week 12 (*P* < 0.001) and became less pronounced gradually between weeks 12 and 18. In the control rats, no significant difference in GCC was observed between rats of different ages (*P* = 0.609), but older rats were found to have thinner INL-ONL layers, photoreceptor layers, and total retina (all *P* < 0.001).

**Figure 4.**
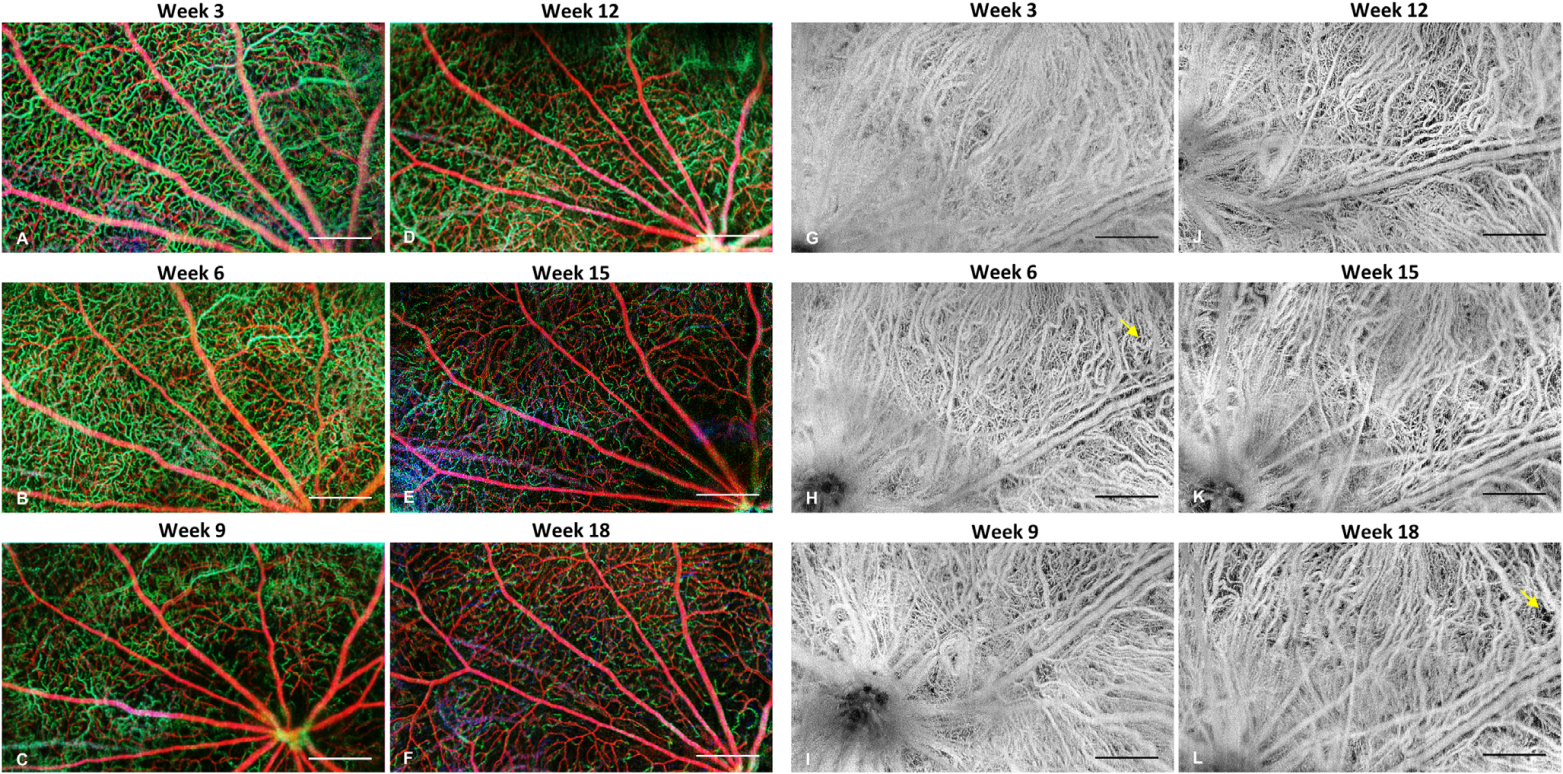
Representative enface angiograms of retina and choroid. (**A–F**) Depth color-coded *enface* angiograms of the inner retina. *Red* represents superficial vascular plexus, and *green* represents deep capillary plexus. (**G–L**) Enface angiograms of the choroidal plexus. *Yellow arrows* indicated the location with choriocapillaris loss from week 6 to week 18. Scale bar = 500 µm.

**Figure 5.**
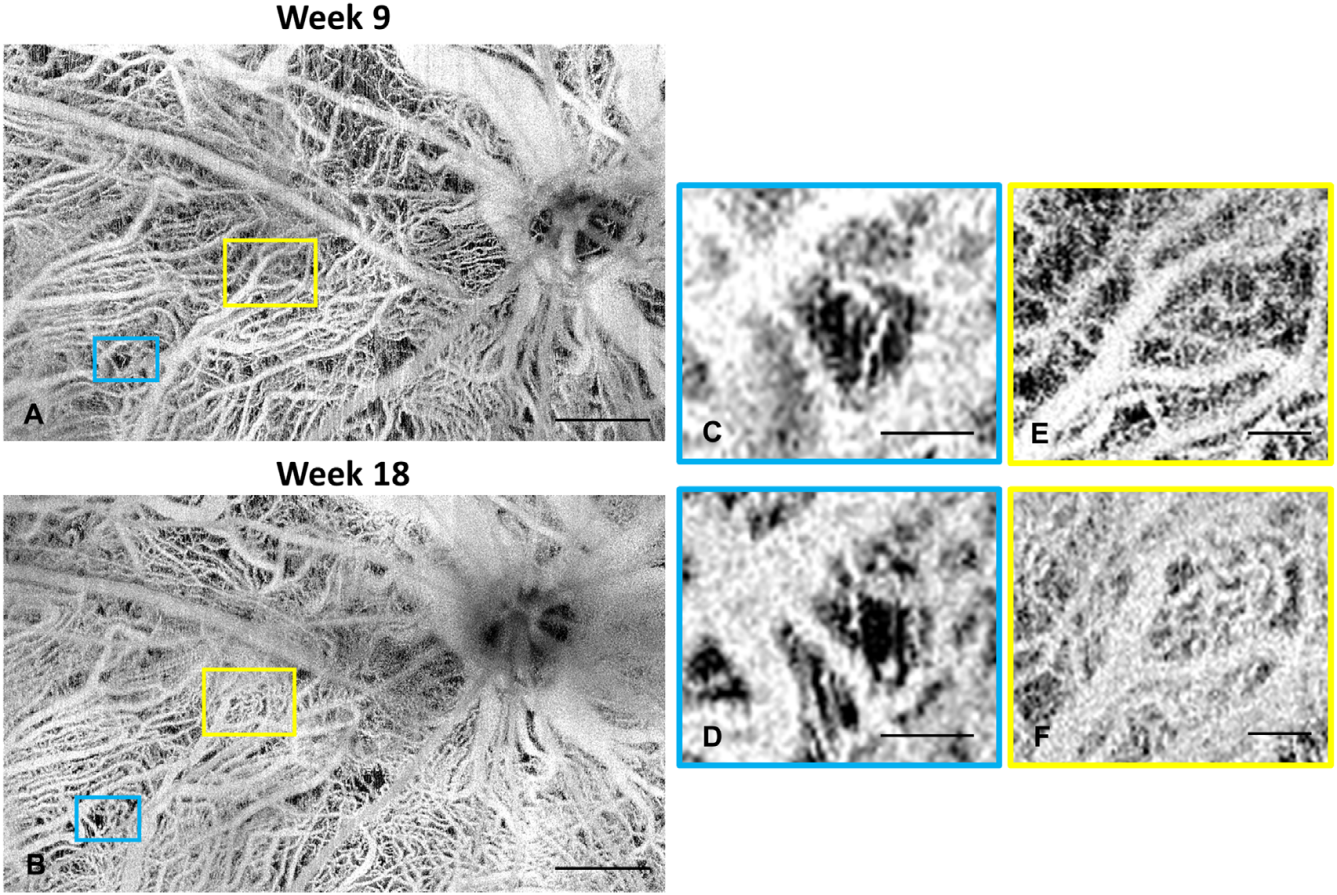
Representative enface angiograms showing choriocapillaris dropout and neovascularization. (**A,B**) enface choroidal angiograms of an RCS rat at weeks 9 and 18. Scale bar = 500 µm. (**C,D**) zoomed in view showing choriocapillaris dropout. (**E,F**) zoomed in view showing neovascularization. Scale bar in **A,B** = 500 µm. Scale bar in the zoomed in figures = 100 µm.

Figure 3 shows the time course of the enface angiograms from the retinal plexuses and the choroid plexus from week 3 to week 18, acquired from a right eye and a left eye of the same rat, respectively. The ONHs were aligned to the inferior-nasal corner for the right eye and inferior-temporal corner for the left eye. In Figures 3A to 3F, the SVP vasculature is depicted in red and the DCP vasculature is depicted in green. Gradual decrease of the DCP was observed while the superficial vasculature didn't show any significant change. The choroid plexus was relatively blurred on week 3 because of the scattering at the RPE layer and became clearer along with dystrophy of RPE. Figure 4 demonstrates representative *enface* choroidal angiograms from another rat showing choroidal vessel changes from week 9 to 18. Zoomed in figures show the locations of choriocapillaris dropout (Figs. 4C, 4D) and neovascularization (Figs. 4E, 4F). The values of the quantitative vascular measures are summarized in Figure 5^6^. In RCS rats there was no change in the OCTA signal derived vessel index in SVP over time (*P* = 0.756). By contrast, the vessel index in the DCP showed a decrease over the study period (*P* trend < 0.001). It decreased strongly from week 3 to week 6 (*P* < 0.001), and more gradually until week 18. For the choroidal plexus, there was an initial increase of the image sharpness from week 3 to 9 (both *P* < 0.030). The sharpness stabilized afterwards with a gradual decline (all *P* > 0.162). The vascular metrics of control rats showed no age dependence (all *P* > 0.05).

**Figure 6.**
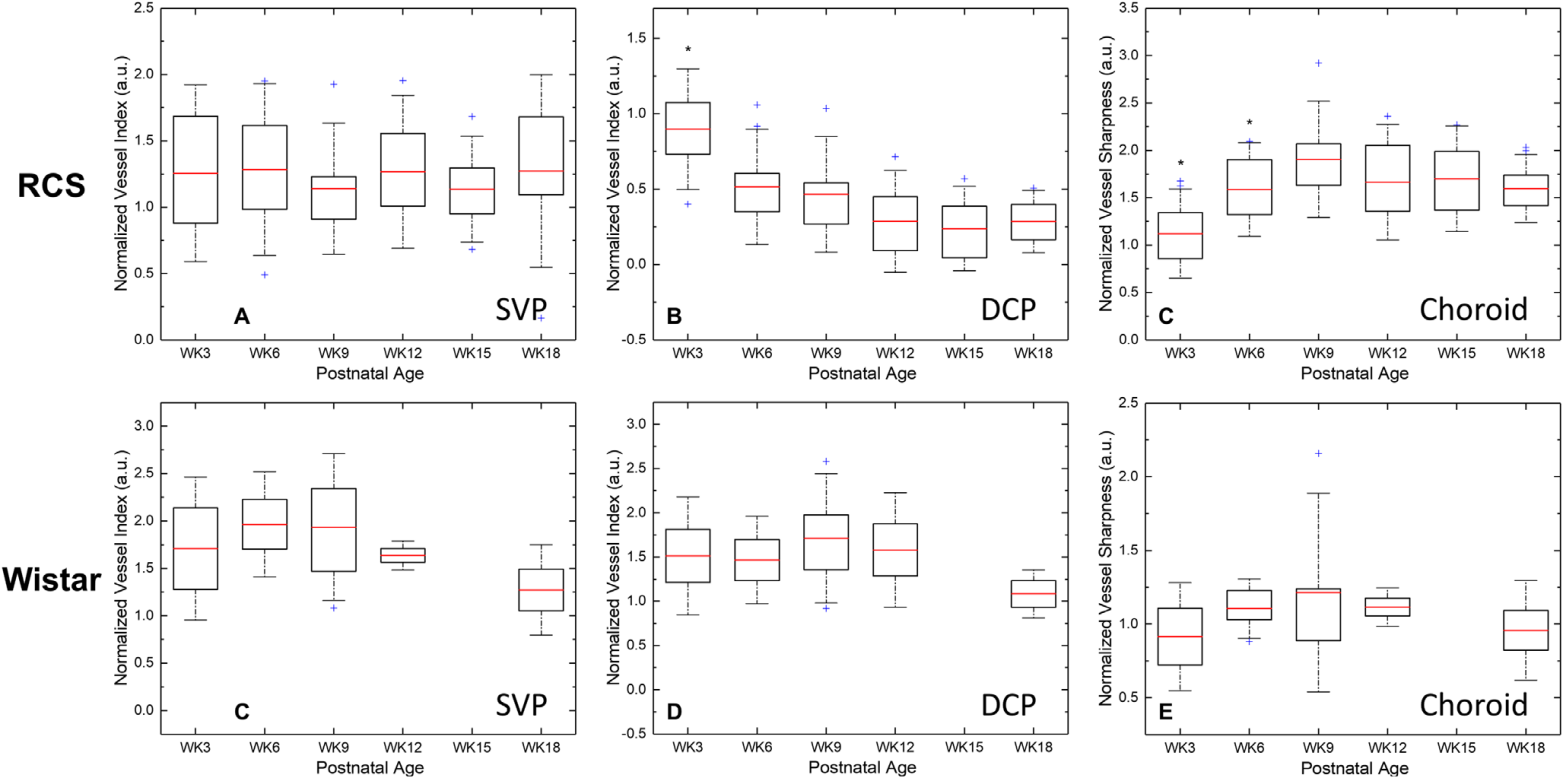
Time course of retinal and choroidal vasculature changes in RCS and age-matched Wistar rats. Normalized vessel index of the inner retinal vasculature and normalized vessel sharpness index of the choroidal vasculature in RCS rats (**A–C**) and age-matched Wistar rats (**D,E**). *Significant difference from the proceeding measurement. *P* < 0.05.

**Figure 7.**
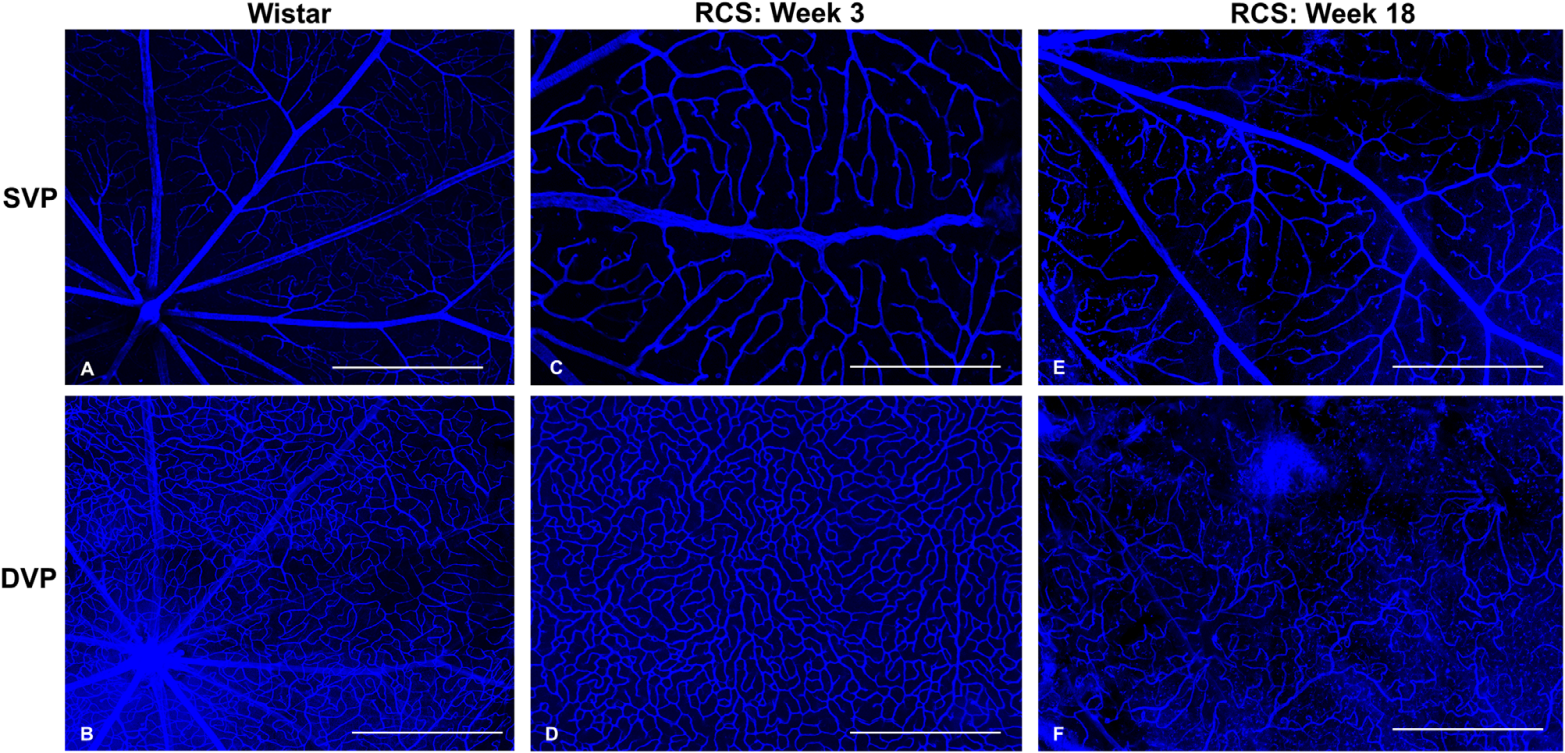
Example of whole-mount histologic images of one Wistar rat and two RCS rats at age 3 and 18 weeks. Scale bar = 500 µm.

The immunochemical results are presented in Figure 7. SVP and DCP of a control rat (age six weeks) and two RCS rats (weeks 3 and 18) are shown. The RCS rat retina vasculature was still under development on week 3, showing irregular vessel branching in SVP. In SVP, vascular abnormalities including moderate neovascularization (Fig. 7D) were noticed at week 18, but in general no significant capillary dropout was seen. A reduction of capillaries in the DCP was clearly observed from weeks 3 to 18.

## **Discussion**

This study provides unique data on the time course of morphologic and vascular changes in the RCS rats. We found a significant decrease in retinal thickness in the inner and the outer retina. In addition, we observed that DCP vessels showed a significant reduction in the RCS rats, whereas the SVP was unaffected. The retinal capillary dropout observed in this study confirmed the results from the previous literature.^33^^,^^51^ Specifically, in one study using FA, the capillary dropout was shown after week 14,^51^ and in another study using whole mount histology, the dropout was firstly observed at week 8 and became more pronounced between week 10-15.^33^ However, neither of the studies reported the capillary plexuses separately. By contrast, OCTA offers the plexus selectivity due to its depth-resolved nature. In previous studies, vessel leakage was observed at different ages,^25^^,^^33^^,^^51^^–^^53^ and the appearance of blood leakage in the dye-injection based imaging modalities confounds the detection and quantification of the capillary dropout. On the contrary, vessel leakage does not modify contrast in the OCTA images and thus has minimal effect on capillary dropout detection.

May et al.^27^^,^^54^ used scanning electron microscopy to image the choriocapillaris of the RCS rats of different ages and reported that choriocapillaris rarefication^27^ and neovascularization^54^ were first observed at month 6. In these studies, however, rats were not studied between months 3 and 6. Our study supports these previous results and further defines the choriocapillaris dropout to occur around week 18. Imaging of the choriocapillaris with OCTA remains challenging, which is mainly due to the highly scattering properties of the RPE layer and the requirements of high lateral resolution and sampling density. In human subjects, the choroidal plexus can only be clearly delineated in severe cases with absence of RPE layer, such as geographic atrophy or panretinal photocoagulation. Our results demonstrate clear visualization of the choriocapillaris from week 6 onwards, when the morphology of the RPE layer was unchanged from the OCT cross-sectional scans. The main source of light scattering in the RPE, melanin granules, had a strong effect on reflectivity in the OCT images, but is susceptible to factors like ocular cloudiness and retinal vessels.^55^ Therefore melanin alteration with RCS degeneration was difficult to be detected in OCT image per se: polarization sensitive OCT may be utilized to visualize the depolarization property of the melanin granules and to provide an extra contrast channel to RPE.^56^ In our study, the sharpness of the choroidal layer underneath RPE could reflect the scattering nature of the RPE, and the latency between RPE degeneration and resultant choriocapillaris dropout could provide a time window to study therapeutic solutions to choroidal vasculopathy. The loss of RPE in the RCS model does, however, prevent a clear analysis of choroidal changes as assessed with OCTA, because it is difficult to decide whether changes as observed in the angiograms are disease-related or simply reflect altered scattering properties due to RPE loss.

Progressive retinal layer thinning with photoreceptor degeneration in RCS rats is well documented using histology and OCT previously.^22^^,^^25^^,^^31^^,^^57^ Recently, Ryals et al.^22^ conducted a longitudinal study using pigmented RCS rats and found the appearance of the hyperreflective debris layer to start from day 30 onward. Notably RCS rats with pigmented eye or pink eye shared a lot of dystrophy similarities. Moreover, the OPL layer was still distinguishable on day 36 but lost its visibility on day 45. These findings are in keeping with our results on PR/debris layer and OPL indicating that the visibility transition happens between week 3 and 6. Furthermore, the gradual thinning of GCC in RCS rats is, to our knowledge, first reported here. The gradual disruption of retinal structure could disrupt the ganglion cell axon bundles which causes subsequent ganglion cell loss.^58^

The present study allows for direct comparison of morphological and vascular changes in RCS rats over time. As such it can also provide insight into the sequence of tissue loss and vessel loss. Interestingly, SVP was not affected despite the gradual thinning of GCC in RCS rats. This indicates that the loss of ganglion cells in this model is not a consequence of vascular insufficiency and that a 12% decrease in GCC does not necessarily lead to a detectable vascular loss at the SVP. This is of relevance for our understanding in diseases such as glaucoma, where there is an ongoing discussion on whether the reduction of blood flow and loss of vessels is a cause or consequence of ganglion cell loss.^59^^–^^64^

Thinner INL-ONL and total retinal thickness with age in control rats is in keeping with previous report on age-related retinal anatomic changes.^65^ Decrease of the nuclear layer thickness until week 12 observed here is related to the growth of the retina area, and Nadal-Nicolás et al.^65^ reported a fast growth of retinal area and progressive neuron layer thinning between day 21 and day 97 in albino rats. Moreover, photoreceptors are culled in retinal developmental phase^66^; however, to our knowledge there is no evidence of correlation between photoreceptor culling and photoreceptor thickness change.

There are some limitations in this study. First, we did not have a control group followed longitudinally, and instead we performed cross sectional measurements in control animals to characterize age dependent changes. Second, the morphological image at week 3 already showed the abnormalities of the photoreceptor layer. A prior change might happen before week 3, and although imaging pups younger than week 3 were previous reported,^31^^,^^67^ it is not possible due to our lab regulations and the OCT system used in the present study. Third, OCTA is a modality to detect the regions with moving particles over a certain speed threshold, and yet it is not able to quantify the blood flow or detect the capillaries with very slow flow velocities.^68^ The OCTA decorrelation signal is sensitive to the flow velocity within a small range, and a signal saturation plateau will be reached when the velocity exceeds this threshold. Therefore neither changes of the blood flow velocity in the large blood vessels nor very slow flow in capillaries can be detected with this technology. A comparison study of OCTA and adaptive optics scanning laser ophthalmoscope indicated that indeed OCTA signals are not detected in certain capillaries due to slow flow.^52^ The histologic images from our study showed good agreement with OCTA images obtained from the superficial layer, but the angiograms from the deep plexus seem to miss some vessels, likely due to the slow flow velocity of the deep capillaries. Fourth, the ROI selection for vessel index calculation was manually obtained by one single grader, which may add some selection bias. Finally, the limited axial resolution and unmatched dispersion compromised the visualization of fine retinal layer structure, which may affect the retinal layer thickness measurement.

In summary, we conducted a longitudinal study of the retinal thickness and vascular changes of retina and choroid in an RCS rat model of retinal degeneration using SS-OCTA. The thickness of the total retina as well as GCC, INL-ONL and debris layers decreased over the weeks. Thinning was more pronounced between weeks 3 and 9 and only slightly continued thereafter. The vasculature in the SVP was unchanged in OCTA images but significantly attenuated in the DCP, which was also confirmed by flat-mount immunohistochemistry. Choriocapillaris dropout was seen, and the vessel sharpness increased from weeks 3 to 9, probably because of the dystrophy of RPE. Our findings indicate that in RCS rats the vascular changes are the consequence of neural and tissue loss. In addition, combined OCT and OCTA studies may provide insight into photoreceptor pathology and allow for the evaluation of therapeutic interventions to prevent photoreceptor degeneration.
